# The relative risk of second primary cancers in Switzerland: a population-based retrospective cohort study

**DOI:** 10.1186/s12885-019-6452-0

**Published:** 2020-01-21

**Authors:** Anita Feller, Katarina L. Matthes, Andrea Bordoni, Christine Bouchardy, Jean-Luc Bulliard, Christian Herrmann, Isabelle Konzelmann, Manuela Maspoli, Mohsen Mousavi, Sabine Rohrmann, Katharina Staehelin, Volker Arndt, the NICER Working Group, K. Staehelin, C. Bouchardy, M. Mousavi, J. L. Bulliard, M. Maspoli, M. Mousavi, A. Bordoni, I. Konzelmann, R. Blanc-Moya, S. Rohrmann

**Affiliations:** 1 Foundation National Institute for Cancer Epidemiology and Registration (NICER), University of Zurich, Zurich, Switzerland; 2 Cancer Registry Zurich and Zug, University Hospital Zurich, Zurich, Switzerland; 3 Ticino Cancer Registry, Instituto cantonale di patologia, Locarno, Switzerland; 4 Geneva Cancer Registry, Institute of Global Health, University of Geneva, Geneva, Switzerland; 5 Vaud Cancer Registry, Centre for Primary Care and Public Health (Unisanté), University of Lausanne, Lausanne, Switzerland; 6 Neuchâtel and Jura Cancer Registry, Neuchâtel, Switzerland; 7 Cancer Registry East Switzerland, St. Gallen, Switzerland; 8 Cancer Registry Grison & Glarus, Chur, Switzerland; 9 Valais Cancer Registry, Health Observatory Valais, Sion, Switzerland; 10 Epidemiology, Biostatistics and Prevention Institute, University of Zurich, Zurich, Switzerland; 11 Cancer Registry Basel-Stadt & Basel-Landschaft, Basel, Switzerland; 12 Unit of Cancer Survivorship, Division of Clinical Epidemiology and Aging Research, German Cancer Research Center (DKFZ), Heidelberg, Germany

**Keywords:** Second primary cancer, Relative risk, Retrospective, Cohort study, Switzerland

## Abstract

**Background:**

More people than ever before are currently living with a diagnosis of cancer and the number of people concerned is likely to continue to rise. Cancer survivors are at risk of developing a second primary cancer (SPC). This study aims to investigate the risk of SPC in Switzerland.

**Methods:**

The study cohort included all patients with a first primary cancer recorded in 9 Swiss population-based cancer registries 1981–2009 who had a minimum survival of 6 months, and a potential follow-up until the end of 2014. We calculated standardized incidence ratios (SIR) to estimate relative risks (RR) of SPC in cancer survivors compared with the cancer risk of the general population. SIR were stratified by type of first cancer, sex, age and period of first diagnosis, survival period and site of SPC.

**Results:**

A total of 33,793 SPC were observed in 310,113 cancer patients. Both male (SIR 1.18, 95%CI 1.16–1.19) and female (SIR 1.20, 95%CI 1.18–1.22) cancer survivors had an elevated risk of developing a SPC. Risk estimates varied substantially according to type of first cancer and were highest in patients initially diagnosed with cancer of the oral cavity and pharynx, Hodgkin lymphoma, laryngeal, oesophageal, or lung cancer. Age-stratified analyses revealed a tendency towards higher RR in patients first diagnosed at younger ages. Stratified by survival period, risk estimates showed a rising trend with increasing time from the initial diagnosis. We observed strong associations between particular types of first and SPC, i.e. cancer types sharing common risk factors such as smoking or alcohol consumption (e.g. repeated cancer of the oral cavity and pharynx (SIR_males_ 20.12, 95%CI 17.91–22.33; SIR_females_ 37.87, 95%CI 30.27–45.48).

**Conclusion:**

Swiss cancer survivors have an increased risk of developing a SPC compared to the general population, particularly patients first diagnosed before age 50 and those surviving more than 10 years. Cancer patients should remain under continued surveillance not only for recurrent cancers but also for new cancers. Some first and SPCs share lifestyle associated risk factors making it important to promote healthier lifestyles in both the general population and cancer survivors.

## Background

More people than ever before are currently living with a diagnosis of cancer and the number of people concerned is likely to continue to rise [1–3]. In the US, for example, 3.6 million cancer survivors were alive in 1975. As of January 2019, the number rose to 16.9 million and that number is expected to increase to 26.1 million by 2040 [4]. Apart from total population growth [5], this trend results from an increase in life expectancy [6], population ageing [5], and continuing improvements in survival linked to better cancer detection and improvement of treatments [7, 8]. A consequence of surviving cancer is the risk of being diagnosed with a second primary cancer (SPC). Cancer survivors might be especially prone to develop a new primary cancer due to various reasons including common etiologic risk factors (i.e. environmental exposures, genetics, lifestyle choices) and late effects of cancer treatment [9]. SPC is not only a particularly difficult event for patients but could be also an important prognostic factor among cancer survivors [10].

Several studies outside of Switzerland reported that cancer survivors have an increased risk for being diagnosed with a new primary cancer compared to the general population [11–17]. However, risk estimates vary strongly by country/region and time period investigated. An older study from Denmark [18], for example, observed a slightly elevated risk of SPC exclusively for those surviving 30 year or more after first diagnosis whereas a recent French study found similar and substantially elevated risks across survival periods [13].

Apparent discrepancies in risk estimates might be partly explained by methodological differences between studies (e.g. definition of metachronous primary cancers, length of follow-up) and/or problems within studies (e.g. incomplete case ascertainment). However, due to potential or known differences between countries and regions (e.g. environmental exposure, standard treatment regimens) and their populations (e.g. risk behaviour, genetic susceptibility), SPC need individual investigations by country or region of interest. The only systematic investigation of risks of SPC in Switzerland dates back to 1993 using data from a single canton cancer registry [19]. A more recent publication covering less than 15% of the Swiss population presented frequencies of first and SPC but no risk estimates of SPC [14]. Therefore, this study aims to investigate the risk of SPC in Switzerland including data of all cantonal cancer registries eligible for this study.

## Methods

Data on cancer diagnoses and vital status information were obtained from Swiss cantonal cancer registries with at least 15 years of consecutive incidence data between 1981 and 2014 (Basel-Stadt/Basel-Landschaft (BS/BL) (1981–2011), Geneva (1981–2014), Grison (1989–2014), Glarus (1992–2014), Neuchâtel (1981–2014), St. Gallen-Appenzell (1981–2014), Ticino (1996–2014), Valais (1989–2014), Vaud (1981–2014) and Zurich (1981–2014)). The national population coverage for this study by calendar year varied from 47.3 to 58.1%. Completeness of case ascertainment in Switzerland is overall high across cancer registries [20]. The proportion of death certificate only cases (DCO) and microscopically verified cases for the registries under study was overall 1.3 and 88.6%, respectively. Nevertheless, in some cancer registries, a systematic and complete linkage of incidence cases against mortality data was not carried out for all calendar years [21]. After exclusion of the affected incidence data, the overall proportion of DCO slightly increased to 1.6%.

A SPC was defined as first subsequent primary cancer occurring at least 6 months after the first cancer [22]. We identified primary cancers based on the rules defined by the International Association of Cancer Registries (IACR), International Association for Research on Cancer (IARC) and the European Network of Cancer Registries (ENCR) [23].

Vital status information was collected by active (verification of vital status with the cantonal and/or community inhabitant control offices) and passive follow-up (matching registered cancer cases with mortality data of the Federal Statistical Office). Due to the lack of a unique patient identifier in the federal mortality data, passive linkage was done using probabilistic matching algorithms. Proportion of cases with incomplete follow-up (i.e. last sign of life before 31st December 2014. (BS/BL: 2011) with no SPC) was 15.7% and ranged by registry from 0 to 24.1%. The variation in follow-up completeness is due to several causes such as the proportion of migrant population, and cantonal organization of cancer registration with different financial resources and legal frameworks. In some cantons, for example, individual inquiry letters have to be sent to inhabitant control offices whereas in other cantons electronic and automatized solutions are well established [24].

Incomplete vital status information artificially decreases follow-up times among patients without SPC leading to an overestimation of SPC risks [25]. We addressed incompleteness of vital status follow-up using multiple imputation methods. We created 25 complete datasets. For each dataset, the analyses were performed separately and merged afterwards using Rubin’s Rules [26]. Incomplete follow-up times were imputed by sex and cancer type using left censored regressions with time period between diagnosis and last follow-up as lower limit. The imputation models included the following predictors: both age at time of diagnosis and incidence period as linear and quadratic term, and language region of residence (French-Italian, German) as categorical variable. Based on imputed follow-up times, corresponding follow-up dates were calculated censoring at 31st of December 2014 (BS/BL: 31st of December 2011). Language region of residence was added to the models due to known regional differences in lifestyle-associated risk factors (i.e. alcohol and tobacco consumption [27]), screening behaviour [28, 29] and treatment regimens [30].

The final study cohort included all patients first diagnosed with an invasive cancer (excluding non-melanoma skin cancer) between 1981 and 2009 (BS/BL: 2006) allowing for a minimum of 5 years after first diagnosis to determine the presence of a SPC. Patients with synchronous primary cancers (cancers diagnosed within 6 months after first diagnosis) and patients with less than 6 months of follow-up were excluded from the main analyses.

We calculated standardized incidence ratios (SIR) (indirect standardization) to estimate relative risks (RR) of metachronous SPC in cancer survivors compared with the cancer risk of the general population [13, 31]. SIR were calculated for all cancers combined (ICD-10 C00-C97 excl. C44) and for 23 common cancer types (19 for males and 21 for females). In addition, the we stratified risk calculations by age at first diagnosis (0–49 years, 50–64 years, 65 years and over), time of first diagnosis (1981–1989, 1990–1999, 2000–2009), and follow-up interval (6 months to less than 1 year, 1 year to less than 5 years, 5 years to less than 10 years, 10 years or longer). Analyses by period of first diagnosis were restricted to 10 years of follow-up to enhance comparability across time. For the five initial cancer types showing the highest risks of developing a SPC and for all first cancer types combined, the RR of SPC were analysed stratified by type of SPC. Person-years at risk were calculated from the 6th month after diagnosis. The comparison cohort (general population) consisted of all permanent residents living between 1981 and 2014 in the catchment area of the included cantonal cancer registries. To calculate the expected number of SPC in the cohort of cancer survivors, we used sex, age- and calendar year-specific incidence rates (5-year age categories) of the general population [17]. Confidence intervals at the 95% level (95%CI) for the SIR were produced assuming a Poisson distribution [32]. 95%CI not including 1 were categorized as significant at p-level < 0.05 [33]. We did not account for multiple comparisons. Significant results have to be interpreted exploratorily.

To investigate the potential effect of detection bias, we additionally calculated the RR of SPC for the time period 0-6th month (synchronous tumours) and for total risks including the first 6 months after initial diagnosis for risk calculations (sensitivity analyses).

In our analyses, we adjusted for age and calendar period, therefore improved survival/aging itself does not increase risk estimates. However, as long-term survivors might be affected by additional risk factors (e.g. long-term and late effects of treatment), we additionally estimated 5-year survival proportions by sex and cancer site using the Kaplan-Meier method.

The multiple imputation of, as well as the calculations of, background risks were carried out using Stata/MP version 15.0 (STAT Corp., TX USA). All further analyses were performed in R (Version 3.4.3).

## Results

The characteristics of the study cohort are presented in Table 1. A total of 33,793 SPC occurred in 310,113 cancer patients over 2,390,410 person-years at risk (median follow-up 6.0 years, interquartile range 2.1 to 10.9 years per person). Almost 40% of SPC were diagnosed between 1 and 5 years after first diagnosis, and nearly one third each were diagnosed 5 to 10 years after first diagnosis or afterwards. The five most common SPC were prostate cancer (28.5%), colorectal cancer (14.2%), cancer of the oral cavity and pharynx (8.5%), bladder cancer (7.3%) and melanoma (6.4%) in males, and breast cancer (36.8%), colorectal cancer (11.7%), cancer of the corpus uteri (9.4%), melanoma (7.2%) and non-Hodgkin lymphoma (3.6%) in females.

**Table 1:** Characteristics of the study cohort

	First Primary Cancers		Second Primary Cancers	
	N	%	N	%
Sex				
Males	155,214	50.1	19,811	58.6
Females	154,899	49.9	13,982	41.4
Age at diagnosis				
15–49 years	54,930	17.7	1250	3.7
50–64 years	93,939	30.3	6708	19.9
65 years and over	161,244	52.0	25,835	76.5
Period of diagnosis				
1981–1984	27,063	8.7	411	1.2
1985–1989	39,184	12.6	1971	5.8
1990–1994	47,624	15.4	3364	10.0
1995–1999	57,764	18.6	4724	14.0
2000–2004	67,145	21.7	6566	19.4
2005–2009	71,333	23.0	8635	25.6
2010–2014	n.a*		8122	24.0
Follow-up interval				
6 month to less than 1 year	n.a.**		2098	6.2
1 years to less than 5 years	n.a.**		12,532	37.1
5 years to less than 10 years	n.a.**		10,011	29.6
10 years and longer	n.a.**		9152	27.1
Type of primary cancer				
Cavity & Pharynx (C00-C14)	8465	2.7	1565	4.6
Oesophagus (C15)	3055	1.0	785	2.3
Stomach (C16)	7663	2.5	1056	3.1
Colorectal (C18-C20)	35,949	11.6	4353	12.9
Extrahepatic Bile Ducts (C22)	2780	0.9	768	2.3
Pancreas (C25)	4534	1.5	1267	3.7
Larynx (C32)	2575	0.8	367	1.1
Lung (C33-C34)	24,539	7.9	5152	15.2
Melanoma (C43)	17,794	5.7	1662	4.9
Breast female (C50)	59,453	19.2	2597	7.7
Cervix uteri (C53)	4080	1.3	125	0.4
Corpus uteri & NOS (C54-C55)	9710	3.1	876	2.6
Ovary (C56)	6067	2.0	530	1.6
Prostate (C61)	48,311	15.6	3694	10.9
Testis (C62)	4912	1.6	36	0.1
Kidney (C64)	6210	2.0	812	2.4
Bladder (C67)	9801	3.2	1387	4.1
Brain & Central Nerves (C70-C72)	4072	1.3	403	1.2
Thyroid (C73)	4360	1.4	414	1.2
Hodgkin Lymphoma (C81)	2481	0.8	123	0.4
non-Hodgkin Lymphoma (C82-C86, C96)	11,340	3.7	1301	3.8
Multiple Myeloma (C90)	4298	1.4	487	1.4
Leukemia (C91-C95)	7933	2.6	1062	3.1
Others	19,731	6.4	2971	8.8
All cancers	310,113	100.0	33,793	100.0

* n.a.: not applicable; the study cohort includes first primary cancers of the period 1981–2009

**n.a.: not applicable; follow-up interval refers to the interval between first and second primary cancer

### Relative risk of second primary cancers by type of first primary cancer and sex

**Fig. 1:**
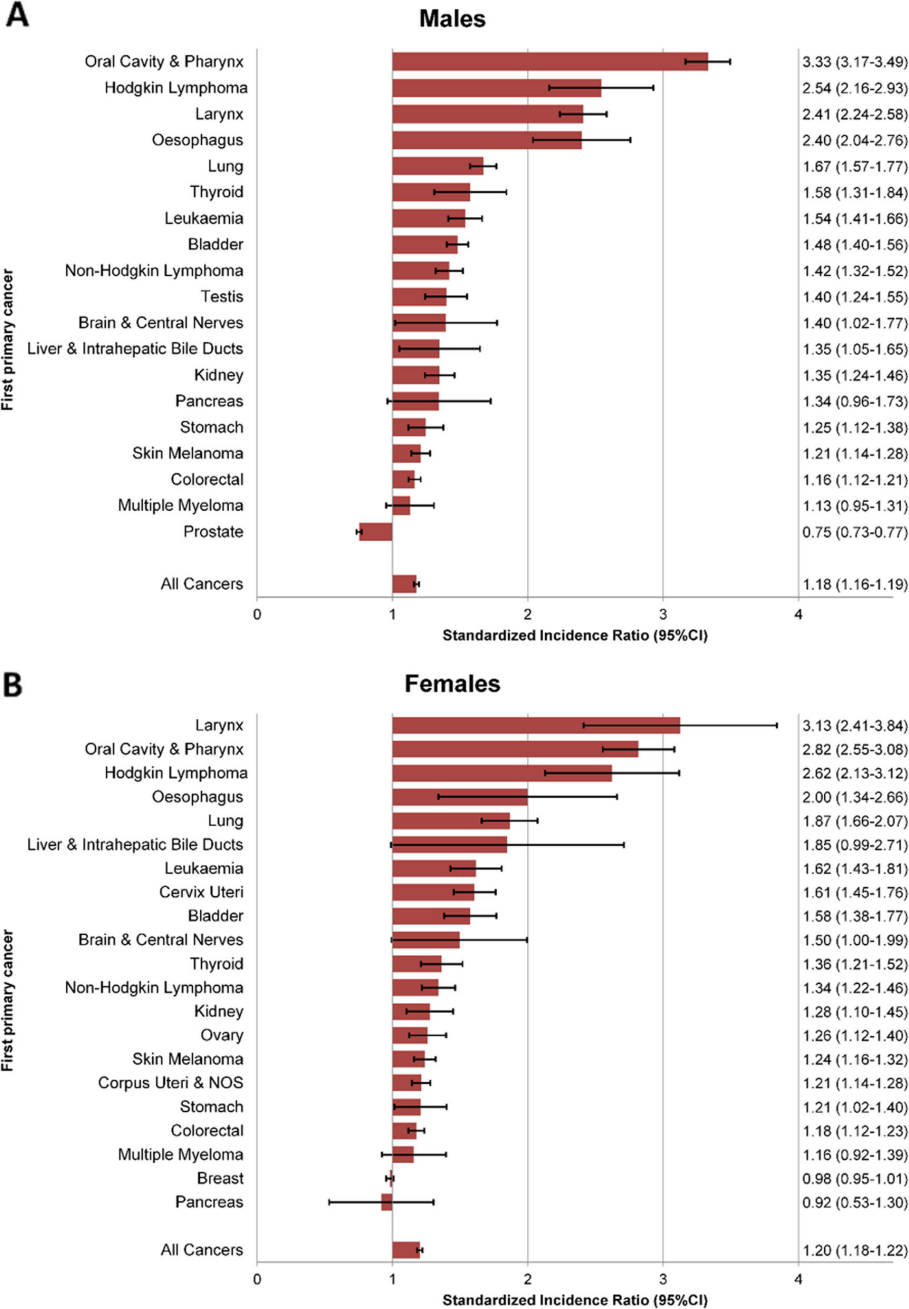
Relative risk of second primary cancers by type of first primary cancer and sex. **a** Males, **b** Females. 95%CI: 95%-confidence interval. Relative risks were estimated by standardized incidence ratios

Overall, both male (SIR 1.18, 95%CI 1.16–1.19) and female (SIR 1.20, 95%CI 1.18–1.22) cancer survivors had an elevated risk of developing a SPC compared with the general population (Fig. 1). In males, 18 out of 19 first primary cancer types investigated were associated with an increased risk for SPC. The RR was statistically significant for all cancer types with the notable exception of pancreatic cancer (SIR 1.34, 95%CI 0.96–1.73) and multiple myeloma (SIR 1.13, 95%CI 0.95–1.31). A significantly reduced risk was observed for prostate cancer (SIR 0.75, 95%CI 0.73–0.77) (Fig. 1), which remained after including tumours diagnosed within the first 6 months after initial diagnosis (SIR 0.80, 95%CI 0.78–0.82) (Additional file 1: Figure S1). In females, the risk of SPC was increased in 19 out of 21 cancer sites, though not reaching statistical significance for multiple myeloma (SIR 1.16, 95%CI 0.92–1.39). The highest SIR were observed for cancers of the oral cavity and pharynx, Hodgkin lymphoma, laryngeal cancer, oesophageal cancer, and lung cancer. For female breast cancer, the observed risk of SPC was similar to the general population (SIR 0.98, 95%CI 0.95–1.01).

Including synchronous tumours (Additional file 1: Table S1 and Figure S1) resulted in higher risk estimates in both sexes (males: SIR 1.32, 95%CI 1.31–1.34; females: SIR 1.29, 95%CI 1.27–1.31) and for all cancer types investigated. A major shift from reduced (main analyses, Fig. 1) to increased risk (sensitivity analyses, Additional file 1: Figure S1) was only observed for female pancreatic cancer. For all other initial cancer types, we observed low to moderate increases, but consistent directions in risk estimates.

The lowest 5-year survival in both sexes were observed for pancreatic (< 5%) and liver cancer (< 10%) (Additional file 1: Table S2). For most initial cancers, women had improved survival compared to men resulting in an overall 5-year survival of 42.2 and 53.0% in males and females, respectively.

### Relative risk of second primary cancer by type of first primary cancer and age at diagnosis

Overall, the risk of SPC compared with the general population was elevated for each of the three age-groups, and tended to decrease with increasing age at first diagnosis (Table 2).

**Table 2:** Relative risk of second primary cancer by site of first primary cancer and age at first diagnosis

	0–49 years				50–64 years				65+ years			
First primary cancer	O	E	SIR	95%CI	O	E	SIR	95%CI	O	E	SIR	95%CI
Oral Cavity & Pharynx	403	61	**6.61**	**(5.94–7.28)**	1103	293	**3.76**	**(3.54–3.99)**	665	324	**2.05**	**(1.89–2.21)**
Oesophagus	30	4	**8.03**	**(4.80–11.25)**	118	41	**2.89**	**(2.33–3.44)**	96	61	**1.57**	**(1.24–1.91)**
Stomach	49	27	**1.79**	**(1.24–2.34)**	205	144	**1.43**	**(1.22–1.63)**	317	292	1.09	(0.96–1.21)
Colorectal	251	139	**1.81**	**(1.58–2.04)**	1376	1086	**1.27**	**(1.20–1.34)**	2814	2576	**1.09**	**(1.05–1.13)**
Liver & Intrahepatic Bile Ducts	17	3	**5.39**	**(2.40–8.37)**	44	29	**1.50**	**(1.01–1.99)**	62	54	1.16	(0.85–1.47)
Pancreas	4	3	1.14	(0.00–2.65)	37	23	**1.61**	**(1.03–2.18)**	51	53	0.97	(0.68–1.26)
Larynx	91	26	**3.56**	**(2.78–4.35)**	470	160	**2.94**	**(2.66–3.21)**	332	176	**1.88**	**(1.67–2.09)**
Lung	120	40	**3.00**	**(2.43–3.57)**	671	339	**1.98**	**(1.82–2.13)**	769	533	**1.44**	**(1.34–1.55)**
Skin Melanoma	386	291	**1.33**	**(1.19–1.46)**	807	668	**1.21**	**(1.12–1.29)**	1083	904	**1.20**	**(1.12–1.27)**
Breast female	748	766	0.98	(0.90–1.05)	2041	2023	1.01	(0.96–1.05)	2356	2456	0.96	(0.92–1.00)
Cervix Uteri	169	104	**1.63**	**(1.37–1.88)**	163	93	**1.75**	**(1.47–2.04)**	126	88	**1.43**	**(1.17–1.70)**
Corpus Uteri & NOS	90	54	**1.66**	**(1.29–2.03)**	581	460	**1.26**	**(1.16–1.37)**	650	576	**1.13**	**(1.04–1.22)**
Ovary	74	48	**1.56**	**(1.17–1.94)**	155	119	**1.30**	**(1.08–1.51)**	138	124	1.11	(0.92–1.31)
Prostate	17	15	1.15	(0.51–1.79)	1269	1656	0.77	(0.72–0.81)	4352	5806	0.75	(0.73–0.77)
Testis	239	172	**1.39**	**(1.21–1.58)**	86	64	**1.34**	**(1.03–1.64)**	22	13	1.75	(0.91–2.59)
Kidney	71	42	**1.69**	**(1.27–2.12)**	331	240	**1.38**	**(1.23–1.54)**	464	370	**1.25**	**(1.14–1.37)**
Bladder	68	32	**2.10**	**(1.56–2.64)**	588	352	**1.67**	**(1.53–1.81)**	1087	781	**1.39**	**(1.31–1.48)**
Brain & Central Nerves	51	25	**2.05**	**(1.43–2.66)**	43	35	1.24	(0.83–1.65)	19	19	0.99	(0.48–1.50)
Thyroid	152	102	**1.49**	**(1.24–1.74)**	191	129	**1.48**	**(1.26–1.70)**	158	121	**1.31**	**(1.09–1.53)**
Hodgkin Lymphoma	174	53	**3.31**	**(2.79–3.83)**	91	40	**2.27**	**(1.77–2.77)**	58	33	**1.77**	**(1.27–2.26)**
Non Hodgkin Lymphoma	162	84	**1.92**	**(1.61–2.23)**	472	337	**1.40**	**(1.27–1.53)**	689	532	**1.30**	**(1.20–1.40)**
Multiple Myeloma	14	11	1.30	(0.50–2.11)	85	75	1.14	(0.88–1.40)	194	171	1.13	(0.96–1.30)
Leukaemia	96	40	**2.43**	**(1.91–2.95)**	334	196	**1.70**	**(1.51–1.89)**	528	377	**1.40**	**(1.28–1.52)**
All Cancers	3783	2256	**1.68**	**(1.62–1.73)**	11,920	9014	**1.32**	**(1.30–1.35)**	18,090	17,190	**1.05**	**(1.04–1.07)**

O: number of observed cases

E: number of expected cases

SIR: standardized incidence ratio

95%CI: 95% confidence intervals

Significantly elevated risks across all age-groups were observed following cancer of the oral cavity and pharynx, oesophageal cancer, colorectal cancer, laryngeal cancer, lung cancer, melanoma, cervical cancer, cancer of the corpus uteri, kidney cancer, bladder cancer, thyroid cancer, Hodgkin lymphoma and leukaemia. Risk were greater among patient who had primary cancer at youngest age for oral cavity, esophagus, colorectal, liver, lung cancers and Hodgkin and non-Hodgkin lymphoma.

For female breast cancer, risk estimates of SPC across all age-groups were comparable to those of the general population (Table 2). Multiple myeloma patients had increased risks across age-groups, albeit not reaching statistical significance. For prostate cancer patients, a reduced risk of SPC was observed for men aged 50 years and above, but not for men diagnosed before the age of 50 years. For the remaining cancer types (stomach, liver, pancreas, ovary, testicles) risks were significantly increased for at least one age-group (< 50 years and/or 50–64 years at initial diagnosis).

### Relative risk of second primary cancers by type of first primary cancer and survival period

The risk of SPC for all initial cancers combined was significantly increased during each survival period showing a rising trend with increasing time since initial diagnosis (Table 3). In addition, significantly elevated risks across all survival periods were observed following cancers of the oral cavity and pharynx, oesopaghus,larynx, lung, kidney and bladder. From 10 years on after initial diagnosis, all cancer sites exhibited significantly elevated RR except pancreatic cancer, multiple myeloma and prostate cancer. Of note, prostate cancer survivors had significantly lower risk of subsequent cancer than the general population. For breast cancer survivors, the risk of developing a SPC was comparable to that of the general population up to 10 years after diagnosis, but increased thereafter.

**Table 3:** Relative risk of second primary cancer by type of first primary cancer and follow-up period

	6 months to 1 year				1 year to 5 years				5 years to 10 years				Over 10 years			
First primary cancer	O	E	SIR	95%CI	O	E	SIR	95%CI	O	E	SIR	95%CI	O	E	SIR	95%CI
Oral Cavity & Pharynx	166	51	**3.28**	**(2.75–3.80)**	971	274	**3.54**	**(3.31–3.77)**	636	200	**3.18**	**(2.93–3.44)**	398	154	**2.59**	**(2.33–2.86)**
Oesophagus	30	17	**1.78**	**(1.06–2.49)**	131	52	**2.51**	**(2.06–2.97)**	47	24	**1.97**	**(1.35–2.59)**	36	13	**2.82**	**(1.79–3.84)**
Stomach	46	47	0.98	(0.67–1.30)	205	188	1.09	(0.93–1.25)	163	122	**1.34**	**(1.12–1.55)**	157	106	**1.48**	**(1.24–1.73)**
Colorectal	280	260	1.08	(0.95–1.21)	1671	1523	**1.10**	**(1.04–1.15)**	1332	1118	**1.19**	**(1.13–1.26)**	1158	900	**1.29**	**(1.21–1.36)**
Liver & Intrahepatic Bile Ducts	20	16	1.26	(0.62–1.89)	58	49	1.19	(0.85–1.52)	28	16	**1.77**	**(1.03–2.52)**	17	5	**3.13**	**(1.40–4.87)**
Pancreas	18	21	0.87	(0.40–1.33)	45	39	1.15	(0.78–1.52)	16	12	1.30	(0.56–2.05)	13	7	1.86	(0.66–3.06)
Larynx	53	20	**2.61**	**(1.84–3.37)**	361	130	**2.77**	**(2.47–3.06)**	256	110	**2.34**	**(2.04–2.63)**	223	102	**2.19**	**(1.89–2.50)**
Lung	201	136	**1.47**	**(1.26–1.69)**	642	420	**1.53**	**(1.41–1.65)**	411	214	**1.92**	**(1.73–2.11)**	306	143	**2.15**	**(1.90–2.40)**
Skin Melanoma	113	89	**1.28**	**(1.03–1.52)**	709	631	**1.12**	**(1.04–1.21)**	687	561	**1.22**	**(1.13–1.32)**	767	583	**1.32**	**(1.22–1.41)**
Breast female	197	252	0.78	(0.67–0.90)	1532	1807	0.85	(0.80–0.89)	1632	1617	1.01	(0.96–1.06)	1784	1570	**1.14**	**(1.08–1.19)**
Cervix Uteri	16	12	1.31	(0.56–2.06)	108	75	**1.45**	**(1.16–1.74)**	124	73	**1.69**	**(1.38–2.01)**	210	125	**1.68**	**(1.44–1.92)**
Corpus Uteri & NOS	46	49	0.95	(0.65–1.25)	386	335	**1.15**	**(1.03–1.27)**	359	320	1.12	(1.00–1.24)	530	386	**1.37**	**(1.25–1.49)**
Ovary	20	23	0.86	(0.43–1.30)	105	113	0.93	(0.74–1.12)	102	75	**1.36**	**(1.08–1.65)**	140	80	**1.75**	**(1.44–2.05)**
Prostate	348	509	0.68	(0.61–0.76)	2423	3409	0.71	(0.68–0.74)	1868	2461	0.76	(0.72–0.79)	999	1098	0.91	(0.85–0.97)
Testis	7	5	1.55	(0.10–3.00)	63	39	**1.60**	**(1.17–2.03)**	70	53	1.33	(0.99–1.67)	207	152	**1.36**	**(1.17–1.56)**
Kidney	65	40	**1.64**	**(1.21–2.07)**	318	251	**1.27**	**(1.12–1.41)**	243	198	**1.23**	**(1.07–1.39)**	240	164	**1.47**	**(1.27–1.66)**
Bladder	150	85	**1.77**	**(1.47–2.06)**	679	476	**1.43**	**(1.32–1.54)**	483	338	**1.43**	**(1.30–1.56)**	431	266	**1.62**	**(1.46–1.78)**
Brain & Central Nerves	15	10	1.52	(0.62–2.43)	38	30	1.27	(0.82–1.72)	25	20	1.26	(0.70–1.82)	35	19	**1.83**	**(1.15–2.50)**
Thyroid	20	14	1.46	(0.73–2.20)	151	103	**1.46**	**(1.22–1.71)**	125	104	1.20	(0.98–1.42)	205	131	**1.57**	**(1.34–1.80)**
Hodgkin Lymphoma	8	5	1.53	(0.21–2.85)	99	34	**2.89**	**(2.28–3.49)**	70	33	**2.09**	**(1.56–2.62)**	146	52	**2.78**	**(2.30–3.26)**
Non Hodgkin Lymphoma	81	64	1.26	(0.97–1.56)	494	388	**1.27**	**(1.16–1.39)**	401	286	**1.40**	**(1.26–1.54)**	347	215	**1.62**	**(1.44–1.79)**
Multiple Myeloma	21	28	0.75	(0.38–1.12)	145	140	1.04	(0.86–1.21)	91	63	**1.44**	**(1.13–1.76)**	36	26	1.38	(0.88–1.88)
Leukaemia	51	45	1.14	(0.80–1.48)	971	266	**1.43**	**(1.29–1.58)**	308	183	**1.68**	**(1.49–1.88)**	218	119	**1.83**	**(1.57–2.08)**
All Cancers	2098	1901	**1.10**	**(1.06–1.15)**	12,532	11,297	**1.11**	**(1.09–1.13)**	10,011	8556	**1.17**	**(1.15–1.19)**	9152	6706	**1.36**	**(1.34–1.39)**

O: number of observed cases

E: number of expected cases

SIR: standardized incidence ratio

95%CI: 95% confidence intervals

### Relative risk of second primary cancer by type of first primary cancer and time period of first diagnosis

For all initial cancers combined andmost cancer sites, no clear trends in risk estimates were observed by time period of first diagnosis (Table 4). However, among patients initially diagnosed with a cancer of the larynx, corpus uteri, bladder or thyroid those diagnosed more recently tended to have a higher risk than those diagnosed earlier. For all initial cancers combined, significantly increased risks were observed for the first (1981–1989: SIR 1.21, 95%CI 1.07–1.36) and last period (2000–2009: SIR 1.17, 95% CI 1.05–1.28), but not for the intermediate period (1990–1999: SIR 1.14, 95%CI 1.00–1.28). However, to allow comparisons that are more consistent across time, these analyses were restricted to 10 years of follow-up, excluding 9152 SPCs and 565,995 person-years from calculation.

**Table 4:** Relative risk of second primary cancer by type of first primary cancer and time period

	1981–1989				1990–1999				2000–2009			
First primary cancer	O	E	SIR	95%CI	O	E	SIR	95%CI	O	E	SIR	95%CI
Oral Cavity & Pharynx	385	115	**3.36**	**(2.89–3.82)**	681	190	**3.58**	**(3.21–3.95)**	707	210	**3.37**	**(3.05–3.70)**
Oesophagus	32	11	**2.93**	**(1.79–4.08)**	79	31	**2.54**	**(1.93–3.15)**	97	50	**1.95**	**(1.52–2.38)**
Stomach	117	100	1.18	(0.90–1.45)	165	134	1.24	(0.98–1.49)	132	112	1.18	(0.92–1.44)
Colorectal	761	628	**1.22**	**(1.01–1.43)**	1183	1028	1.16	(0.96–1.36)	1339	1137	**1.18**	**(1.00–1.37)**
Liver & Intrahepatic Bile Ducts	9	5	1.79	(0.35–3.23)	25	22	1.12	(0.62–1.63)	72	52	**1.37**	**(1.03–1.72)**
Pancreas	15	11	1.34	(0.53–2.15)	22	23	0.98	(0.50–1.45)	42	37	1.14	(0.75–1.53)
Larynx	170	73	**2.32**	**(1.92–2.71)**	247	94	**2.63**	**(2.27–3.00)**	253	89	**2.83**	**(2.44–3.23)**
Lung	291	171	**1.70**	**(1.49–1.91)**	433	266	**1.63**	**(1.46–1.80)**	530	325	**1.63**	**(1.47–1.79)**
Skin Melanoma	264	212	**1.25**	**(1.05–1.44)**	536	441	**1.22**	**(1.05–1.39)**	709	593	**1.20**	**(1.04–1.36)**
Breast female	721	756	0.96	(0.81–1.10)	1205	1301	0.93	(0.81–1.05)	1435	1516	0.95	(0.85–1.05)
Cervix Uteri	97	57	**1.71**	**(1.34–2.08)**	86	61	**1.42**	**(1.08–1.75)**	65	40	**1.63**	**(1.18–2.07)**
Corpus Uteri & NOS	197	193	1.02	(0.86–1.19)	285	251	1.14	(0.96–1.31)	309	243	**1.27**	**(1.08–1.47)**
Ovary	57	48	1.18	(0.84–1.52)	100	78	1.28	(1.00–1.56)	70	81	0.86	(0.64–1.09)
Prostate	608	858	0.71	(0.59–0.84)	1544	2203	0.70	(0.60–0.81)	2487	3137	0.79	(0.72–0.87)
Testis	31	22	1.43	(0.86–1.99)	54	38	**1.43**	**(1.01–1.85)**	55	37	**1.49**	**(1.06–1.92)**
Kidney	123	95	**1.29**	**(1.03–1.55)**	207	186	1.11	(0.94–1.29)	296	197	**1.50**	**(1.28–1.72)**
Bladder	328	242	**1.36**	**(1.14–1.58)**	514	352	**1.47**	**(1.22–1.71)**	470	277	**1.70**	**(1.43–1.98)**
Brain & Central Nerves	15	10	1.43	(0.58–2.28)	24	22	1.11	(0.60–1.62)	39	27	1.43	(0.93–1.93)
Thyroid	58	48	1.22	(0.87–1.57)	102	76	**1.35**	**(1.06–1.64)**	136	95	**1.44**	**(1.18–1.70)**
Hodgkin Lymphoma	41	17	**2.40**	**(1.58–3.21)**	59	26	**2.27**	**(1.63–2.90)**	77	29	**2.63**	**(1.99–3.27)**
Non Hodgkin Lymphoma	185	138	**1.34**	**(1.10–1.59)**	347	265	**1.31**	**(1.13–1.50)**	444	318	**1.40**	**(1.21–1.59)**
Multiple Myeloma	58	45	1.28	(0.91–1.66)	83	77	1.08	(0.81–1.35)	116	103	1.12	(0.89–1.36)
Leukaemia	167	106	**1.58**	**(1.27–1.89)**	246	168	**1.47**	**(1.21–1.73)**	327	206	**1.59**	**(1.35–1.84)**
All Cancers	5052	4172	**1.21**	**(1.07–1.36)**	8752	7679	1.14	(1.00–1.28)	10,837	9308	**1.17**	**(1.05–1.28)**

O: number of observed cases

E: number of expected cases

SIR: standardized incidence ratio

95%CI: 95% confidence intervals

### Relative risk of second primary cancers by type of first and second primary cancer and sex

**Fig. 2:**
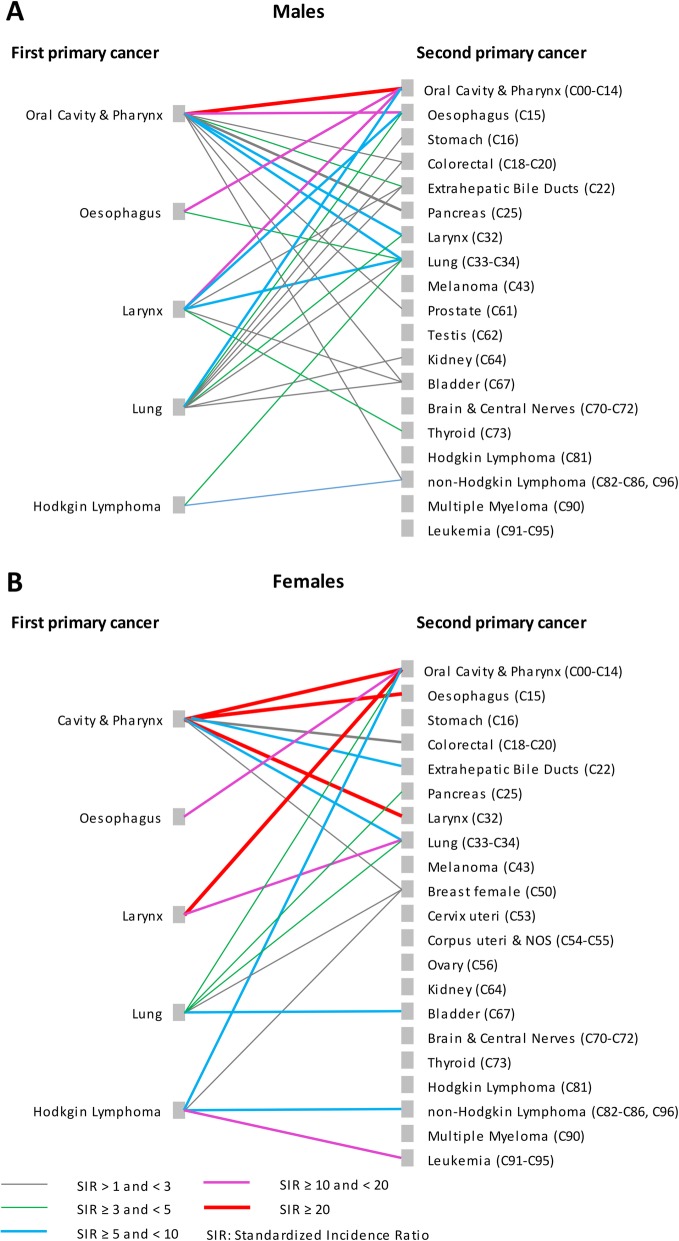
Relative risk of second primary cancer for selected first primary cancer sites by type of second primary cancer and sex. **a** Males, **b** Females. Relative risks were estimated by standardized incidence ratios

Figure 2 presents the RR estimates (limited to statistically significant results) by type of SPC for the five first primary cancers with the highest RR of SPC. Complete lists of calculated risk estimates are supplied in Additional file 1: Table S3, S4, S5, S6 and S7.

The strongest combinations of initial cancer and SPC (SIR > = 20) were observed for:

- cancer of the oral cavity and pharynx – oral cavity and pharynx (both sexes)
- cancer of the oral cavity and pharynx – laryngeal cancer (females)
- cancer of the oral cavity and pharynx – oesophageal cancer (females)
- laryngeal cancer – oral cavity and pharynx (females).

Overall, an initial diagnosis with cancer of the oral cavity and pharynx was associated with significantly increased risks for 10 (out of 19) and 7 (out of 21) types of SPC in males and females, respectively. Similarly, lung cancer showed elevated risks for 9 specific SPC in males and 5 in females. Corresponding figures for initial laryngeal cancer and oesophageal cancer were 6 and 2, and 2 and 1 specific SPC types in males and females, respectively.

In patients initially diagnosed with Hodgkin lymphoma significantly elevated risks estimates were observed for second non-Hodgkin lymphoma and lung cancer in males, and second leukemia, cancer of the oral cavity and pharynx, bladder and breast in females.

For all initial cancers combined, we observed significantly increased risks for 17 out of 19 specific SPC types in males and 17 out of 21 specific SPC types in females, ranging overall from SIR 1.18 (95%CI 1.13–1.22, colorectal cancer in males) to SIR 2.53 (95%CI 2.38–2.68, cancer of the oral cavity and pharynx in males) (Table 5). In contrast, male cancer survivors had a significantly reduced risk of developing second prostate cancer (SIR 0.67, 95%CI 0.65–0.70) and female cancer survivors a reduced risk of developing second breast (SIR 0.74, 95%CI 0.71–0.77) and cervical cancers (SIR 0.82, 95%CI 0.66–0.98).

**Table 5:** Relative risk of second primary cancer for any first primary cancer by type of second primary cancer and sex

	Males				Females			
Second primary cancer	O	E	SIR	95%CI	O	E	SIR	95%CI
Oral Cavity & Pharynx	1136	449.60	**2.53**	**(2.38–2.68)**	429	189.05	**2.27**	**(2.05–2.49)**
Oesophagus	566	293.21	**1.93**	**(1.76–2.10)**	219	96.60	**2.27**	**(1.95–2.59)**
Stomach	670	528.41	**1.27**	**(1.17–1.37)**	386	299.80	**1.29**	**(1.15–1.42)**
Colorectal	2430	2066.81	**1.18**	**(1.13–1.22)**	1923	1470.15	**1.31**	**(1.25–1.37)**
Liver & Intrahepatic Bile Ducts	576	453.56	**1.27**	**(1.16–1.38)**	192	137.66	**1.39**	**(1.19–1.60)**
Pancreas	669	490.60	**1.36**	**(1.26–1.47)**	598	469.70	**1.27**	**(1.17–1.38)**
Larynx	317	179.31	**1.77**	**(1.56–1.97)**	50	26.04	**1.92**	**(1.31–5.53)**
Lung	3551	2262.39	**1.57**	**(1.52–1.62)**	1601	891.66	**1.80**	**(1.71–1.88)**
Skin Melanoma	926	670.74	**1.38**	**(1.29–1.47)**	736	513.03	**1.43**	**(1.33–1.54)**
Breast female	–	–	–	–	2597	3515.70	0.74	(0.71–0.77)
Cervix Uteri	–	–	–	–	125	152.49	0.82	(0.66–0.98)
Corpus Uteri & NOS	–	–	–	–	876	674.95	**1.30**	**(1.21–1.39)**
Ovary	–	–	–	–	530	437.46	**1.21**	**(1.10–1.32)**
Prostate	3694	5486.76	0.67	(0.65–0.70)	–	–	–	–
Testis	36	49.08	0.73	(0.45–1.02)	–	–	–	–
Kidney	537	396.46	**1.35**	**(1.23–1.47)**	275	196.64	**1.40**	**(1.22–1.57)**
Bladder	1053	855.25	**1.23**	**(1.16–1.31)**	334	238.59	**1.40**	**(1.24–1.56)**
Brain & Central Nerves	242	184.26	**1.31**	**(1.14–1.49)**	161	139.94	1.15	(0.96–1.34)
Thyroid	143	60.76	**2.35**	**(1.93–2.77)**	271	156.18	**1.74**	**(1.52–1.95)**
Hodgkin Lymphoma	67	39.21	**1.71**	**(1.25–2.17)**	56	28.16	**1.99**	**(1.39–2.58)**
Non Hodgkin Lymphoma	733	539.77	**1.36**	**(1.26–1.46)**	568	440.42	**1.29**	**(1.18–1.40)**
Multiple Myeloma	291	231.10	**1.26**	**(1.11–1.41)**	196	188.29	1.04	(0.88–1.20)
Leukaemia	590	428.02	**1.38**	**(1.26–1.49)**	472	283.12	**1.67**	**(1.51–1.82)**
All Cancers	18,811	16,827.19	**1.18**	**(1.16–1.19)**	13,982	11,631.97	**1.20**	**(1.18–1.22)**

O: number of observed cases

E: number of expected cases

SIR: standardized incidence ratio

95%CI: 95% confidence intervals

## Discussion

### Summary of main findings

Overall, Swiss cancer survivors had a moderate excess risk of SPC (about 20% in both sexes) compared to the general population. SPC risks varied substantially according to site of first primary cancer and were highest among patients initially diagnosed with cancer of the oral cavity and pharynx, larynx, oesophagus, lung and Hodgkin lymphoma. Prostate cancer survivors had a significantly reduced risk of developing a SPC and no excess risk of SPC was found in female breast cancer patients, apart beyond 10 years after first diagnosis. Overall and for most initial cancers, a tendency towards higher RR was observed in patients first diagnosed at younger ages. Stratified by survival period, risk estimates increased with time since first diagnosis. Our data also further confirmed strong associations between particular types of first and SPC, mainly for cancers caused by smoking. For example, Swiss males and females initially diagnosed with cancer of the oral cavity and pharynx had a 20-fold and almost 40-fold increased risk of being diagnosed with a second cancer of the oral cavity and pharynx, and a 16-fold and almost 30-fold increased risk of being confronted with a second oesophageal cancer, respectively.

### Discussion in the context of the literature

In line with our Swiss results, studies conducted in other high-income countries reported an increased risk for cancer survivors of being diagnosed with SPC compared to the cancer risk within the general population [12–17, 34]. However, overall increased risks of subsequent cancers were not systematically observed for all regions or countries and time periods investigated. A recent cancer registry study from Austria, for example, observed increased risks for single cancer sites, but no overall increased risk [35]. An Italian population-based study reported an overall significantly reduced risk (SIR 0.87) although the population size covered (< 2.5 million inhabitants in total) and the length of follow-up (mean: 2.5 years, < 50,000 person-years of follow-up) were relatively limited [36].

Excess risk of SPC is usually explained by common aetiologic risk factors (i.e. lifestyle, environmental exposures and genetics) and/or late effects of cancer treatment (i.e. radiotherapy and chemotherapy). Smoking and alcohol consumption are clearly associated with an elevated risk of various cancers [37], and the multiplicative effect of both factors can substantially exceed the risk from either factor alone for some *cancer sites* [37*]*. Consequently, smokers/regular drinkers diagnosed with a smoking/alcohol-associated cancer have an elevated risk of developing a second smoking/alcohol associated cancer [11, 38]. Overall, smoking and alcohol-associated cancers accounted for a substantial proportion of first and second primary cancers in our study population. Many *cancers* are known to *have a multifactorial etiology and some cancer treatments are known to be carcinogenic.* For example, known risk factors for breast cancer include age, genetics, being overweight, exposure to estrogen, alcohol consumption and others. For other cancers, such as Hodgkin lymphoma, the aetiology of disease remains largely unknown [39]. Due to the lack of data on risk factors and treatment in our study, underlying mechanisms could not be investigated and are therefore speculative.

In line with other studies outside of Switzerland [35, 40], we observed a significantly reduced risk of SPC for prostate cancer survivors. Prostate cancer is the most common cancer in males, leading to high numbers of expected cases. However, our risk of subsequent prostate cancer might be overestimated. Overall, more than 50% of patients with first prostate cancer are treated with surgery including radical prostatectomy [41]. In Switzerland, prostate cancer incidence rates have recently decreased after a sustained increase up to 2002, most likely due to changes in screening and work-up practices [42]. Higher socioeconomic position (SEP) has been shown to be associated with more frequent prostate-specific antigen (PSA) screening, earlier diagnosis [28, 43] and higher prostate cancer incidence compared to men with lower SEP. [44, 45] In addition, men of higher SEP are generally more health conscious than the general male population [46] and thus less likely to be exposed to cancer risk such as cigarette smoking and alcohol consumption.

Similarly, breast cancer is the most common cancer in females leading to high number of expected second breast cancers. However, women treated with mastectomy are not at risk for ipsilateral breast cancer [47]. Concerning SEP and mammography use, the Swiss findings are mixed [48, 49], but we recently reported that women of higher SEP compared to women of lower SEP are more likely to be diagnosed at an early stage [50]. In our present study, breast cancer survivors in general had a similar risk of SPC than the general female population, although an increased risk of SPC was observed in the youngest age-group (< 50 years at time of diagnosis). The latter finding might be related to factors such as cancer-predisposition genes [51] or more aggressive treatment schemes [52].

In line with other studies [11, 13, 17, 35], we found a consistent pattern of higher risks of SPC for young-onset patients. Notably, longer residual life expectancy itself is insufficient to explain our results as we calculated age-adjusted risks. However, improved survival in younger patients may increase the likelihood of experiencing adverse long-term treatment effects. Further, younger patients may receive more aggressive treatment [53, 54] and might differ from older patients in various respects such as genetic predisposition, risk factor patterns, etc.

SPC may occur months or years after the first cancer was diagnosed. However, SPC caused by first cancer treatment are expected to occur many years after the first diagnosis [55]. For therapy-associated solid tumours, the latency period is typically 10 years or more. Treatment-related leukemia occurs with a shorter latency, peaking between 5 to 10 years after diagnosis [55]. In our study, the risk of SPC was significantly increased during each follow-up period with a substantial increase at 5 to 10 years and over 10 years post-diagnosis. This is in contrast to studies from Australia and the US, which reported increased risks of SPC but no relevant changes by follow-up period.

In the US and Australia, the risk of SPC appears to have increased since the 1980s [56]. However, whether increased radiation exposure and/or changed treatment modalities contributed to this finding is not determined [56]. Interestingly, the rise over time in SPC found for thyroid cancer in the present study largely concur with the advent of growing thyroid cancer incidence most likely related to screening and increased overdiagnosis [57]. Overall, we observed no clear temporal trends after stratification by time period. For all cancer sites combined, we found a significantly increased risk of SPC for the first and last time period, but not for the intermediate one. However, these analyses were restricted to 10 years of follow-up, excluding a substantial number of SPCs and person-years from calculation.

Patterns of first and second primary cancers were further analysed for the five cancers with the highest RR of SPC, out of which four are highly related to smoking (Fig. 2). Our results confirmed mutual increased risks between tobacco-related cancer types [13, 17]. For patients with Hodgkin lymphoma, a cancer of largely unknown aetiology [39], the RR of SPC ranked second in males and third in females. Previous studies have shown that Hodgkin lymphoma is associated with increased risks for both second solid tumours and hematologic malignancies which persist for many decades after treatment [58, 59]. The risk of SPC following Hodgkin lymphoma seems to be influenced by various factors including age at treatment [58, 59], treatment type [58], smoking [60] and family history of cancer [59]. We confirmed an increased risk of second non-Hodgkin lymphoma and leukaemia among Hodgkin lymphoma patients, as well as increased risks for second breast cancer and lung cancer but not for other second malignancies as reported previously [58, 59].

### Strengths and limitations

This is the largest population-based investigation of SPC among cancer survivors in Switzerland, providing the most representative nationwide findings to date. This study was based on data from population-based registries of high quality and high completeness of case ascertainment [20].

However, there are several methodological aspects to point out. First, there are no universally agreed criteria to identify multiple primary cancers. Currently, mainly two definitions of multiple primaries are used, one from the Surveillance Epidemiology and End Results (SEER) Program [61], the other from the IACR and the IARC [62]. Although both definitions take site and histology into account, the definition of the SEER Program are more complex and classify more cases as multiple primary cancers (i.e. cancers diagnosed at the same site, in paired organs and/or with similar histology) than the IACR/IARC definition [63]. A detailed description and comparison of both definitions and rules can be found elsewhere [63]. For this study, we applied the IACR/IARC rules, resulting in more conservative risk estimates than those based on the SEER definition [40]. The impact of these different definitions has been assessed in Scotland, based on data of 10 pooled cancer sites with 5-year follow-up, resulting in a SIR of 0.86 and a SIR of 1.0 when applying the IACR/IARC and SEER rules, respectively. A large difference was reported however for repeated breast cancer in women, of which 79 of 98 subsequent breast cancers were classified as SPC by SEER, but only 1 by IACR/IARC [40]. In addition, there is no common definition of second or metachronous tumours. We used a 6-month delay as cut-off between synchronous and metachronous tumours [22]. However, shorter limits have also been used [13, 16, 17]. Finally, a few authors classified all subsequent tumours as metachronous, irrespective of time lag between first and second cancer diagnosis [64, 65]. Our sensitivity analysis, including primary cancers occurring within 6 months after initial diagnosis, revealed higher risk estimates than our main analyses. Consequently, our estimates are likely to be conservative**.**

The gradual implementation of regional cancer registration in Switzerland means that some SPC may have been wrongly classified as first cancers if the true first cancer was diagnosed prior to the implementation of cancer registration in that region. However, the magnitude of this bias is likely to be small. In France, based on data of a single cancer registry collecting data since 1958, a comparison of RR estimates of SPC obtained over fictional registration periods beginning 10 years, 5 years and 0 years before the study period reported small overestimations of RR of 0.1, 0.6, and 0.6%, respectively.

Although overall completeness of case ascertainment in Switzerland is high, potential underregistration of lymphoid leukemia was consistently observed across cancer registries [20]. For the cancer registry of Basel, potential underregistration was also reported for liver, pancreatic and ovarial cancer; for the cancer registry of Zurich also for non-Hodgkin lymphoma and ovarial, prostate and kidney cancer. At the national level (all registries combined), ovarial cancer, prostate cancer and lymphoid leukemia showed suspicious results regarding completeness. Consequently, it cannot be ruled out that some of the presented estimates are affected by underregistration. However, our results correspond very well to the results published from countries with high completeness of cancer registration, such as Denmark, Finland and Norway [12, 15, 18].

Finally, we cannot exclude chance findings resulting from multiple comparisons, or the possibility that genuine effects have been missed due to low statistical power for some comparisons.

## Conclusions

Swiss cancer survivors have an increased risk of developing a SPC compared to the general population. This risk is especially high among patients first diagnosed before age 50 years and those surviving at least 10 years. Cancer patients should remain under continued surveillance not only for recurrent cancers but also for new primary cancers. First and second cancers that share common lifestyle associated risk factors are rather common and underlines the importance to promote healthier lifestyles in both the general population and cancer survivors.

## Supplementary information

**Additional file 1: Table S1.** Relative risk of synchronous primary tumour (follow-up period 0–6 month). **Table S2.** Median age at diagnosis and 5-year observed survival by cancer site and sex. **Table S3.** Relative risk of second primary cancer following cancer of the oral cavity and pharynx by type of second primary cancer and sex. **Table S4**. Relative risk of second primary cancer following oesophageal cancer by type of second primary cancer and sex. **Table S5.** Relative risk of second primary cancer following laryngeal cancer by type of second primary cancer and sex. **Table S6.** Relative risk of second primary cancer following lung cancer by type of second primary cancer and sex. **Table S7.** Relative risk of second primary cancer following Hodgkin lymphoma by type of second primary cancer and sex. **Figure S1.** Relative risk of second primary cancer by site of first primary cancer and sex including synchronous tumours including the first 6 month after initial diagnosis for risk calculations.

## Data Availability

The datasets used and/or analysed during the current study are available from the corresponding author on reasonable request.

## Abbreviations

95%CI: 95% confidence interval
BS/BL: Basel-Stadt/Basel-Landschaft
DCO: Death certificate only cases
ENCR: European Network of Cancer Registries
IACR: International Association of Cancer Registries
IARC: International Association for Research on Cancer
ICD-10: International Classification of Diseases, 10th revision
PSA: prostate-specific antigen
RR: Relative risk
SEER: Surveillance Epidemiology and End Results Program
SEP: Socioeconomic position
SIR: Standardized incidence ratio
SPC: Second primary cancer
