# Correction to: The relative risk of second primary cancers in Switzerland: a population-based retrospective cohort study

**DOI:** 10.1186/s12885-020-6584-2

**Published:** 2020-02-03

**Authors:** Anita Feller, Katarina L. Matthes, Andrea Bordoni, Christine Bouchardy, Jean-Luc Bulliard, Christian Herrmann, Isabelle Konzelmann, Manuela Maspoli, Mohsen Mousavi, Sabine Rohrmann, Katharina Staehelin, Volker Arndt, the NICER Working Group, K. Staehelin, C. Bouchardy, M. Mousavi, J. L. Bulliard, M. Maspoli, M. Mousavi, A. Bordoni, I. Konzelmann, J. L. Bulliard, R. BlancMoya, S. Rohrmann

**Affiliations:** 1 Foundation National Institute for Cancer Epidemiology and Registration (NICER), University of Zurich, Zurich, Switzerland; 2 Cancer Registry Zurich and Zug, University Hospital Zurich, Zurich, Switzerland; 3 Ticino Cancer Registry, Instituto cantonale di patologia, Locarno, Switzerland; 4 Geneva Cancer Registry, Institute of Global Health, University of Geneva, Geneva, Switzerland; 5 Vaud Cancer Registry, Centre for Primary Care and Public Health (Unisanté), Feller et al. BMC Cancer (2020) 20:51 Page 13 of 15 University of Lausanne, Lausanne, Switzerland; 6 Neuchâtel and Jura Cancer Registry, Neuchâtel, Switzerland; 7 Cancer Registry East Switzerland, St. Gallen, Switzerland; 8 Cancer Registry Grison & Glarus, Chur, Switzerland; 9 Valais Cancer Registry, Health Observatory Valais, Sion, Switzerland; 10 Epidemiology, Biostatistics and Prevention Institute, University of Zurich, Zurich, Switzerland; 11 Cancer Registry Basel-Stadt & Basel-Landschaft, Basel, Switzerland; 12 Unit of Cancer Survivorship, Division of Clinical Epidemiology and Aging Research, German Cancer Research Center (DKFZ), Heidelberg, Germany

**Correction to: BMC Cancer (2020) 20:51**

**https://doi.org/10.1186/s12885-019-6452-0**

Following publication of the original article [1], an error was reported in the author group. The correct author group should read as follows:

Anita Feller^1^*, Katarina L. Matthes^2^, Andrea Bordoni^3^, Christine Bouchardy^4^, Jean-Luc Bulliard^5,6^, Christian Herrmann^7,8^, Isabelle Konzelmann^9^, Manuela Maspoli^6^, Mohsen Mousavi^7,8^, Sabine Rohrmann^2,10^, Katharina Staehelin^11^, Volker Arndt^1,12^, the NICER Working Group.

The original article [1] has been corrected.
