# Supplementary material for: The relative risk of second primary cancers in Switzerland: a population-based retrospective cohort study
Source: BMC Cancer. 2020 Jan 21;20:51. doi: 10.1186/s12885-019-6452-0 (PMC6974968; doi:10.1186/s12885-019-6452-0)
Supplement: Supplementary file 1 — Additional file 1: Table S1. Relative risk of synchronous primary tumour (follow-up period 0–6 month). Table S2. Median age at diagnosis and 5-year observed survival by cancer site and sex. Table S3. Relative risk of second primary cancer following cancer of the oral cavity and pharynx by type of second primary cancer and sex. Table S4. Relative risk of second primary cancer following oesophageal cancer by type of second primary cancer and sex. Table S5. Relative risk of second primary cancer following laryngeal cancer by type of second primary cancer and sex. Table S6. Relative risk of second primary cancer following lung cancer by type of second primary cancer and sex. Table S7. Relative risk of second primary cancer following Hodgkin lymphoma by type of second primary cancer and sex. Figure S1. Relative risk of second primary cancer by site of first primary cancer and sex including synchronous tumours including the first 6 month after initial diagnosis for risk calculations. [file 12885_2019_6452_MOESM1_ESM.docx]

**Additional File 1: Supplementary Material**

**Table S1: Relative risk of synchronous primary tumour (follow-up period 0-6 month)**

|  | **Both sexes** | | | |
| --- | --- | --- | --- | --- |
| **First primary cancer** | O | E | **SIR** | **95%CI** |
| Oral Cavity & Pharynx | 469 | 60 | **7.88** | **(7.14-8.61)** |
| Oesophagus | 154 | 28 | **5.5** | **(4.59-6.42)** |
| Stomach | 197 | 71 | **2.79** | **(2.38-3.20)** |
| Colorectal | 933 | 298 | **3.13** | **(2.92-3.33)** |
| Liver & Intrahepatic Bile Ducts | 123 | 29 | **4.26** | **(3.46-5.06)** |
| Pancreas | 137 | 48 | **2.88** | **(2.37-3.39)** |
| Larynx | 98 | 22 | **4.46** | **(3.52-5.40)** |
| Lung | 765 | 226 | **3.39** | **(3.15-3.64)** |
| Skin Melanoma | 174 | 92 | **1.88** | **(1.59-2.18)** |
| Breast female | 384 | 259 | **1.48** | **(1.33-1.63)** |
| Cervix Uteri | 63 | 14 | **4.61** | **(3.38-5.84)** |
| Corpus Uteri & NOS | 204 | 52 | **3.92** | **(3.36-4.49)** |
| Ovary | 107 | 29 | **3.71** | **(2.96-4.46)** |
| Prostate | 715 | 532 | **1.34** | **(1.24-1.44)** |
| Testis | 9 | 5 | 1.97 | (0.39-3.55) |
| Kidney | 125 | 47 | **2.68** | **(2.19-3.18)** |
| Bladder | 632 | 101 | **6.24** | **(5.74-6.74)** |
| Brain & Central Nerves | 43 | 19 | **2.23** | **(1.50-2.97)** |
| Thyroid | 43 | 15 | **2.86** | **(1.92-3.80)** |
| Hodgkin Lymphoma | 22 | 6 | **3.71** | **(1.94-5.49)** |
| Non Hodgkin Lymphoma | 209 | 76 | **2.76** | **(2.37-3.15)** |
| Multiple Myeloma | 73 | 33 | **2.22** | **(1.67-2.77)** |
| Leukaemia | 154 | 54 | **2.87** | **(2.39-3.35)** |
| **All Cancers** | **6,248** | **2,261** | **2.76** | **(2.69-2.83)** |

O: number of observed cases
E: number of expected cases
SIR: standardized incidence ratio
95%CI: 95% confidence intervals

**Table S2: Median age at diagnosis and 5-year observed survival by cancer site and sex**

|  | **Males** | | |  | **Females** | | |
| --- | --- | --- | --- | --- | --- | --- | --- |
| **Site of first primary cancer** | **Median age at diagnosis** | **5-year**  **observed survival (%)** | **95%CI** |  | **Median age at diagnosis** | **5-year**  **observed survival (%)** | **95%CI** |
| Oral cavity & Pharynx (C00-C14) | 60 | 39.1 | (36.5-41.7) |  | 62 | 52.8 | (49.5-56.0) |
| Oesophagus (C15) | 65 | 11.4 | (2.7-20.1) |  | 72 | 14.1 | (1.1-27.1) |
| Stomach (C16) | 69 | 19.9 | (15.4-24.3) |  | 75 | 21.2 | (16.1-26.3) |
| Colorectal (C18-C20) | 70 | 44.3 | (43.0-45.7) |  | 73 | 46.1 | (44.7-47.5) |
| Extrahepatic Bile Ducts (C22) | 68 | 6.9 | (0.0-16.7) |  | 72 | 7.7 | (0.0-23.7) |
| Pancreas (C25) | 69 | 3.4 | (0.0-17.5) |  | 74 | 3.4 | (0.0-16.9) |
| Larynx (C32) | 62 | 56.2 | (53.1-59.2) |  | 63 | 58.5 | (51.0-66.1) |
| Lung (C33-C34) | 67 | 10.0 | (6.8-13.2) |  | 67 | 13.5 | (9.1-17.9) |
| Melanoma (C43) | 61 | 73.9 | (72.8-75.1) |  | 56 | 83.4 | (82.5-84.2) |
| Breast female (C50) | - | - | - |  | 62 | 74.3 | (73.8-74.7) |
| Cervix uteri (C53) | - | - | - |  | 53 | 62.5 | (60.4-64.7) |
| Corpus uteri & NOS (C54-C55) | - | - | - |  | 67 | 68.6 | (67.4-69.8) |
| Ovary (C56) | - | - | - |  | 66 | 33.4 | (30.3-36.4) |
| Prostate (C61) | 71 | 62.9 | (62.3-63.5) |  | - | - | - |
| Testis (C62) | 34 | 93.9 | (93.2-94.6) |  | - | - | - |
| Kidney (C64) | 66 | 48.4 | (45.7-51.0) |  | 69 | 49.7 | (46.3-53.1) |
| Bladder (C67) | 71 | 44.7 | (42.6-46.9) |  | 75 | 38.3 | (34.2-42.4) |
| Brain & Central Nerves (C70-C72) | 57 | 21.3 | (15.2-27.4) |  | 61 | 19.3 | (12.0-26.7) |
| Thyroid (C73) | 54 | 71.0 | (67.6-74.4) |  | 51 | 82.4 | (80.9-83.8) |
| Hodgkin Lymphoma (C81) | 38 | 77.6 | (75.0-80.2) |  | 34 | 78.9 | (76.0-81.7) |
| non-Hodgkin Lymphoma (C82-C86, C96) | 64 | 49.1 | (46.9-51.3) |  | 69 | 52.6 | (50.5-54.8) |
| Multiple Myeloma (C90) | 69 | 32.8 | (27.6-38.1) |  | 72 | 33.2 | (27.8-38.5) |
| Leukemia (C91-C95) | 65 | 45.7 | (43.0-48.3) |  | 69 | 43.3 | (40.1-46.5) |
|  |  |  |  |  |  |  |  |
| All cancers | 68 | 42.2 | (41.7-42.7) |  | 66 | 53.0 | (52.6-53.5) |

**Table S3: Relative risk of second primary cancer following cancer of the oral cavity and pharynx by type of second primary cancer and sex**

|  | **Males** | | | |  | **Females** | | | |
| --- | --- | --- | --- | --- | --- | --- | --- | --- | --- |
| **Second primary cancer** | O | E | **SIR** | **95%CI** |  | **O** | **E** | **SIR** | **95%CI** |
| Oral Cavity & Pharynx | 343 | 17.05 | **20.12** | **(17.91-22.33)** |  | 108 | 2.85 | **37.87** | **(30.27-45.48)** |
| Oesophagus | 148 | 9.45 | **15.66** | **(12.92-18.39)** |  | 39 | 1.41 | **27.69** | **(17.48-37.91)** |
| Stomach | 24 | 15.81 | 1.52 | (0.83-2.21) |  | 2 | 4.27 | 0.47 | (0.00-1.45) |
| Colorectal | 87 | 60.48 | **1.44** | **(1.11-1.76)** |  | 35 | 21.17 | **1.65** | **(1.04-2.26)** |
| Liver & Intrahepatic Bile Ducts | 52 | 13.96 | **3.72** | **(2.62.4.83)** |  | 10 | 1.99 | **5.02** | **(1.24-8.80)** |
| Pancreas | 29 | 14.30 | **2.03** | **(1.20-2.86)** |  | 11 | 6.70 | 1.64 | (0.47-2.81) |
| Larynx | 64 | 6.50 | **9.84** | **(7.11-12.58)** |  | 12 | 0.40 | **30.29** | **(7.24-53.34)** |
| Lung | 504 | 71.66 | **7.03** | **(6.40-7.68)** |  | 100 | 13.30 | **7.52** | **(5.94-9.09)** |
| Skin Melanoma | 17 | 20.66 | 0.82 | (0.37-1.28) |  | 5 | 7.64 | 0.65 | (0.00-1.41) |
| Breast female | - | - | - | - |  | 81 | 52.98 | **1.53** | **(1.16-1.90)** |
| Cervix Uteri | - | - | - | - |  | 4 | 2.23 | 1.79 | (0.00-4.58) |
| Corpus Uteri & NOS | - | - | - | - |  | 9 | 10.08 | 0.89 | (0.09-1.69) |
| Ovary | - | - | - | - |  | 10 | 6.48 | 1.54 | (0.25-2.84) |
| Prostate | 213 | 158.87 | **1.34** | **(1.15-1.53)** |  | - | - | - | - |
| Testis | 2 | 1.89 | 1.06 | (0.00-3.83) |  | - | - | - | - |
| Kidney | 13 | 12.65 | 1.03 | (0.29-1.77) |  | 4 | 2.86 | 1.40 | (0.00-3.27) |
| Bladder | 41 | 24.21 | **1.69** | **(1.12-2.27)** |  | 8 | 3.40 | 2.35 | (0.33-4.38) |
| Brain & Central Nerves | 5 | 6.39 | 0.78 | (0.00-1.88) |  | 2 | 2.09 | 0.96 | (0.00-3.83) |
| Thyroid | 8 | 2.09 | 3.82 | (0.00-7.64) |  | 4 | 2.36 | 1.70 | (0.00-4.52) |
| Hodgkin Lymphoma | 1 | 1.31 | 0.76 | (0.00-5.41) |  | 1 | 0.42 | 2.41 | (0.00-17.09) |
| Non Hodgkin Lymphoma | 33 | 16.34 | **2.02** | **(1.25-2.79)** |  | 7 | 6.40 | 1.09 | (0.07-2.12) |
| Multiple Myeloma | 8 | 6.77 | 1.18 | (0.00-2.36) |  | 0 | 2.72 | 0.00 | - |
| Leukaemia | 15 | 12.52 | 1.20 | (0.41-1.99) |  | 4 | 4.07 | 0.98 | (0.00-2.62) |
| All Cancers | 1,688 | 506.83 | **3.33** | **(3.17-3.49)** |  | 483 | 171.43 | **2.82** | **(2.55-3.08)** |

O: number of observed cases
E: number of expected cases
SIR: standardized incidence ratio
95%CI: 95% confidence intervals

**Table S4: Relative risk of second primary cancer following oesophageal cancer by type of second primary cancer and sex**

|  | **Males** | | | |  | **Females** | | | |
| --- | --- | --- | --- | --- | --- | --- | --- | --- | --- |
| **Second primary cancer** | O | E | **SIR** | **95%CI** |  | **O** | **E** | **SIR** | **95%CI** |
| Oral Cavity & Pharynx | 48 | **2.60** | **18.47** | **(12.74-24.20)** |  | 7 | 0.38 | **18.61** | **(1.23-36.00)** |
| Oesophagus | 1 | 1.52 | 0.66 | (0.00-4.66) |  | 0 | 0.20 | 0.00 | - |
| Stomach | 9 | 2.41 | 3.74 | (0.74-6.74) |  | 2 | 0.59 | 3.37 | (0.00-10.41) |
| Colorectal | 15 | 9.75 | 1.54 | (0.62-2.45) |  | 1 | 3.01 | 0.33 | (0.00-1.46) |
| Liver & Intrahepatic Bile Ducts | 5 | 2.33 | 2.15 | (0.00.4.62) |  | 1 | 0.29 | 3.48 | (0.00-15.30) |
| Pancreas | 7 | 2.29 | 3.05 | (0.20-5.90) |  | 1 | 0.96 | 1.05 | (0.00-4.60) |
| Larynx | 6 | 1.01 | 5.93 | (0.00-13.20) |  | 1 | 0.05 | 18.67 | (0.00-132.56) |
| Lung | 36 | 11.44 | **3.15** | **(2.00-4.29)** |  | 16 | 1.88 | 8.53 | (0.00-13.42) |
| Skin Melanoma | 2 | 3.40 | 0.59 | (0.00-1.81) |  | 1 | 1.00 | 1.00 | (0.00-4.42) |
| Breast female | - | - | - | - |  | 11 | 7.01 | 1.57 | (0.33-2.81) |
| Cervix Uteri | - | - | - | - |  | 1 | 0.28 | 3.56 | (0.00-19.86) |
| Corpus Uteri & NOS | - | - | - | - |  | 0 | 1.41 | 0.00 | (0.00-2.62) |
| Ovary | - | - | - | - |  | 0 | 0.90 | 0.00 | (0.00-4.10) |
| Prostate | 37 | 26.81 | 1.38 | (0.86-1.90) |  | - | - | - | - |
| Testis | 0 | 0.21 | 0.00 | (0.00-17.89) |  | - | - | - | - |
| Kidney | 5 | 2.03 | 2.46 | (0.00-5.92) |  | 0 | 0.41 | 0.00 | - |
| Bladder | 6 | 3.87 | 1.55 | (0.00-3.14) |  | 2 | 0.49 | 4.11 | (0.00-12.69) |
| Brain & Central Nerves | 2 | 1.01 | 1.98 | (0.00-7.91) |  | 0 | 0.29 | 0.00 | - |
| Thyroid | 2 | 0.32 | 6.33 | (0.00-25.31) |  | 0 | 0.28 | 0.00 | - |
| Hodgkin Lymphoma | 0 | 0.19 | 0.00 | (0.00-0.00) |  | 0 | 0.05 | 0.00 | - |
| Non Hodgkin Lymphoma | 2 | 2.60 | 0.77 | (0.00-2.37) |  | 1 | 0.91 | 1.10 | (0.00-4.85) |
| Multiple Myeloma | 0 | 1.10 | 0.00 | - |  | 0 | 0.40 | 0.00 | - |
| Leukaemia | 3 | 1.99 | 1.51 | (0.00-4.68) |  | 0 | 0.58 | 0.00 | - |
| All Cancers | 1,688 | 506.83 | **3.33** | **(3.17-3.49)** |  | 47 | 23.52 | **2.00** | **(1.34-2.66)** |

O: number of observed cases
E: number of expected cases
SIR: standardized incidence ratio
95%CI: 95% confidence intervals

**Table S5: Relative risk of second primary cancer following laryngeal cancer by type of second primary cancer and sex**

|  | **Males** | | | |  | **Females** | | | |
| --- | --- | --- | --- | --- | --- | --- | --- | --- | --- |
| **Second primary cancer** | O | E | **SIR** | **95%CI** |  | **O** | **E** | **SIR** | **95%CI** |
| Oral Cavity & Pharynx | 124 | 10.23 | **12.12** | **(9.86-14.38)** |  | 20 | 0.47 | **42.21** | **(20.90-63.52)** |
| Oesophagus | 38 | 6.02 | **6.31** | **(3.95-8.67)** |  | 7 | 0.24 | 29.72 | (0.00-62.36) |
| Stomach | 16 | 10.54 | 1.52 | (0.65-2.39) |  | 1 | 0.73 | 1.38 | (0.00-6.07) |
| Colorectal | 53 | 40.17 | 1.32 | (0.93-1.71) |  | 1 | 3.61 | 0.28 | (0.00-1.22) |
| Liver & Intrahepatic Bile Ducts | 20 | 9.08 | **2.20** | **(1.09-3.31)** |  | 1 | 0.34 | 2.95 | (0.00-13.00) |
| Pancreas | 10 | 9.50 | 1.05 | (0.26-1.85) |  | 1 | 1.13 | 0.89 | (0.00-3.90) |
| Larynx | 2 | 4.08 | 0.49 | (0.00-1.96) |  | 0 | 0.07 | 0.00 | (0.00-0.00) |
| Lung | 307 | 47.85 | **6.42** | **(5.67-7.16)** |  | 27 | 2.29 | **11.79** | **(6.76-16.82)** |
| Skin Melanoma | 13 | 12.90 | 1.01 | (0.36-1.66) |  | 3 | 1.25 | 2.41 | (0.00-6.24) |
| Breast female | - | - | - | - |  | 13 | 8.97 | 1.45 | (0.42-2.48) |
| Cervix Uteri | - | - | - | - |  | 0 | 0.38 | 0.00 | (0.00-9.70) |
| Corpus Uteri & NOS | - | - | - | - |  | 2 | 1.79 | 1.12 | (0.00-4.04) |
| Ovary | - | - | - | - |  | 0 | 1.13 | 0.00 | (0.00-3.27) |
| Prostate | 84 | 105.64 | 0.80 | (0.61-0.98) |  | - | - | - | - |
| Testis | 0 | 0.75 | 0.00 | (0.00-4.93) |  | - | - | - | - |
| Kidney | 16 | 8.17 | 1.96 | (0.72-3.20) |  | 3 | 0.50 | 6.04 | (0.00-18.74) |
| Bladder | 37 | 16.51 | **2.24** | **(1.44-3.04)** |  | 3 | 0.58 | 5.21 | (0.00-13.50) |
| Brain & Central Nerves | 3 | 3.94 | 0.76 | (0.00-2.36) |  | 0 | 0.36 | 0.00 | - |
| Thyroid | 4 | 1.23 | 3.24 | (0.00-8.64) |  | 0 | 0.38 | 0.00 | - |
| Hodgkin Lymphoma | 1 | 0.76 | 1.32 | (0.00-9.34) |  | 0 | 0.07 | 0.00 | - |
| Non Hodgkin Lymphoma | 17 | 10.57 | 1.61 | (0.72-2.50) |  | 3 | 1.10 | 2.73 | (0.00-7.07) |
| Multiple Myeloma | 1 | 4.47 | 0.22 | (0.00-1.59) |  | 0 | 0.47 | 0.00 | - |
| Leukaemia | 10 | 8.22 | 1.22 | (0.17-2.26) |  | 0 | 0.69 | 0.00 | - |
| All Cancers | 802 | 332.91 | **2.41** | **(2.24-2.58)** |  | 91 | 29.12 | **3.13** | **(2.41-3.84)** |

O: number of observed cases
E: number of expected cases
SIR: standardized incidence ratio
95%CI: 95% confidence intervals

**Table S6: Relative risk of second primary cancer following lung cancer by type of second primary cancer and sex**

|  | **Males** | | | |  | **Females** | | | |
| --- | --- | --- | --- | --- | --- | --- | --- | --- | --- |
| **Second primary cancer** | O | E | **SIR** | **95%CI** |  | **O** | **E** | **SIR** | **95%CI** |
| Oral Cavity & Pharynx | 112 | 21.52 | **5.21** | **(4.18-6.23)** |  | 14 | 3.12 | **4.49** | **(1.71-7.27)** |
| Oesophagus | 52 | 12.92 | **4.02** | **(2.77-5.28)** |  | 8 | 1.49 | 5.38 | (0.00-10.75) |
| Stomach | 44 | 23.41 | **1.88** | **(1.27-2.49)** |  | 4 | 4.13 | 0.97 | (0.00-2.25) |
| Colorectal | 139 | 88.15 | **1.58** | **(1.30-1.85)** |  | 32 | 22.04 | 1.45 | (0.89-2.01) |
| Liver & Intrahepatic Bile Ducts | 41 | 19.77 | **2.07** | **(1.37-2.77)** |  | 1 | 2.16 | 0.46 | (0.00-2.04) |
| Pancreas | 32 | 20.68 | 1.55 | (0.95-2.15) |  | 22 | 6.96 | **3.16** | **(1.65-3.90)** |
| Larynx | 33 | 8.83 | **3.74** | **(2.22-5.26)** |  | 2 | 0.44 | 4.50 | (0.00-0.00) |
| Lung | 190 | 107.04 | **1.78** | **(1.51-2.04)** |  | 60 | 15.32 | **3.92** | **(2.84-4.99)** |
| Skin Melanoma | 22 | 27.12 | 0.81 | (0.42-1.20) |  | 12 | 8.39 | 1.43 | (0.46-2.40) |
| Breast female | - | - | - | - |  | 85 | 58.68 | **1.45** | **(1.11-1.79)** |
| Cervix Uteri | - | - | - | - |  | 6 | 2.34 | 2.56 | (0.00-5.57) |
| Corpus Uteri & NOS | - | - | - | - |  | 11 | 11.34 | 0.97 | (0.20-1.74) |
| Ovary | - | - | - | - |  | 15 | 7.10 | 2.11 | (0.74-3.48) |
| Prostate | 246 | 230.10 | 1.07 | (0.93-1.21) |  | - | - | - | - |
| Testis | 0 | 1.54 | 0.00 | (0.00-2.40) |  | - | - | - | - |
| Kidney | 33 | 17.81 | **1.85** | **(1.10-2.61)** |  | 6 | 3.14 | 1.91 | (0.00-4.25) |
| Bladder | 100 | 36.37 | **2.75** | **(2.17-3.32)** |  | 13 | 3.47 | **3.75** | **(1.33-6.17)** |
| Brain & Central Nerves | 12 | 8.47 | 1.42 | (0.34-2.49) |  | 2 | 2.37 | 0.84 | (0.00-3.38) |
| Thyroid | 8 | 2.60 | 3.08 | (0.00-6.16) |  | 4 | 2.57 | 1.56 | (0.00-4.15) |
| Hodgkin Lymphoma | 4 | 1.63 | 2.46 | (0.00-9.54) |  | 0 | 0.44 | 0.00 | - |
| Non Hodgkin Lymphoma | 32 | 22.84 | 1.40 | (0.86-1.94) |  | 10 | 6.95 | 1.44 | (0.35-2.53) |
| Multiple Myeloma | 11 | 9.72 | 1.13 | (0.22-2.04) |  | 4 | 2.98 | 1.34 | (0.00-3.58) |
| Leukaemia | 28 | 17.99 | 1.56 | (0.86-2.25) |  | 8 | 4.29 | 1.87 | (0.00-3.73) |
| All Cancers | 1,214 | 726.88 | **1.67** | **(1.57-1.77)** |  | 346 | 185.48 | **1.87** | **(1.66-2.07)** |

O: number of observed cases
E: number of expected cases
SIR: standardized incidence ratio
95%CI: 95% confidence intervals

**Table S7: Relative risk of second primary cancer following Hodgkin lymphoma by type of second primary cancer and sex**

|  | **Males** | | | |  | **Females** | | | |
| --- | --- | --- | --- | --- | --- | --- | --- | --- | --- |
| **Second primary cancer** | O | E | **SIR** | **95%CI** |  | **O** | **E** | **SIR** | **95%CI** |
| Oral Cavity & Pharynx | 11 | 3.29 | 3.34 | (0.96-5.72) |  | 8 | 0.83 | **9.59** | **(1.33-17.84)** |
| Oesophagus | 0 | 1.45 | 0.00 | - |  | 1 | 0.31 | 3.25 | (0.00-23.06) |
| Stomach | 2 | 2.22 | 0.90 | (0.00-2.78) |  | 1 | 0.97 | 1.03 | (0.00-4.53) |
| Colorectal | 14 | 8.38 | 1.67 | (0.64-2.71) |  | 6 | 4.77 | 1.26 | (0.00-2.55) |
| Liver & Intrahepatic Bile Ducts | 8 | 2.10 | 3.81 | (0.53-7.08) |  | 1 | 0.45 | 2.24 | (0.00-9.86) |
| Pancreas | 6 | 2.01 | 2.99 | (0.00-6.05) |  | 4 | 1.39 | 2.89 | (0.00-6.70) |
| Larynx | 5 | 1.08 | 4.62 | (0.00-11.10) |  | 0 | 0.11 | 0.00 | - |
| Lung | 50 | 10.08 | **4.96** | **(3.45-6.47)** |  | 9 | 3.33 | 2.70 | (0.53-4.87) |
| Skin Melanoma | 9 | 4.38 | 2.05 | (0.41-3.70) |  | 8 | 3.36 | 2.38 | (0.33-4.43) |
| Breast female | - | - | - | - |  | 42 | 16.92 | **2.48** | **(1.61-3.36)** |
| Cervix Uteri | - | - | - | - |  | 0 | 1.14 | 0.00 | (0.00-3.23) |
| Corpus Uteri & NOS | - | - | - | - |  | 3 | 2.49 | 1.20 | (0.00-3.52) |
| Ovary | - | - | - | - |  | 1 | 1.77 | 0.57 | (0.00-3.16) |
| Prostate | 25 | 20.12 | 1.24 | (0.65-1.83) |  | - | - | - | - |
| Testis | 1 | 2.39 | 0.42 | (0.00-2.33) |  | - | - | - | - |
| Kidney | 3 | 2.06 | **1.45** | **(1.10-4.51)** |  | 2 | 0.70 | 2.87 | (0.00-11.48) |
| Bladder | 7 | 3.07 | 2.28 | (0.15-4.41) |  | 2 | 0.69 | **2.91** | **(1.33-8.97)** |
| Brain & Central Nerves | 0 | 1.45 | 0.00 | - |  | 1 | 0.72 | 1.39 | (0.00-9.88) |
| Thyroid | 3 | 0.65 | 4.65 | (0.00-14.42) |  | 4 | 1.47 | 2.72 | (0.00-7.25) |
| Hodgkin Lymphoma | 0 | 0.66 | 0.00 | - |  | 0 | 0.38 | 0.00 | - |
| Non-Hodgkin Lymphoma | 21 | 2.93 | **7.17** | **(3.65-10.69)** |  | 13 | 1.71 | 7.61 | (0.69-12.53) |
| Multiple Myeloma | 2 | 0.99 | 2.02 | (0.00-8.06) |  | 1 | 0.61 | 1.65 | (0.00-11.68) |
| Leukaemia | 9 | 2.03 | 4.44 | (0.35-8.54) |  | 11 | 1.05 | **10.50** | **(2.04-18.96)** |
| All Cancers | 195 | 76.70 | **2.54** | **(2.16-2.93)** |  | 128 | 48.81 | **2.62** | **(2.13-3.12)** |

O: number of observed cases
E: number of expected cases
SIR: standardized incidence ratio
95%CI: 95% confidence intervals

**
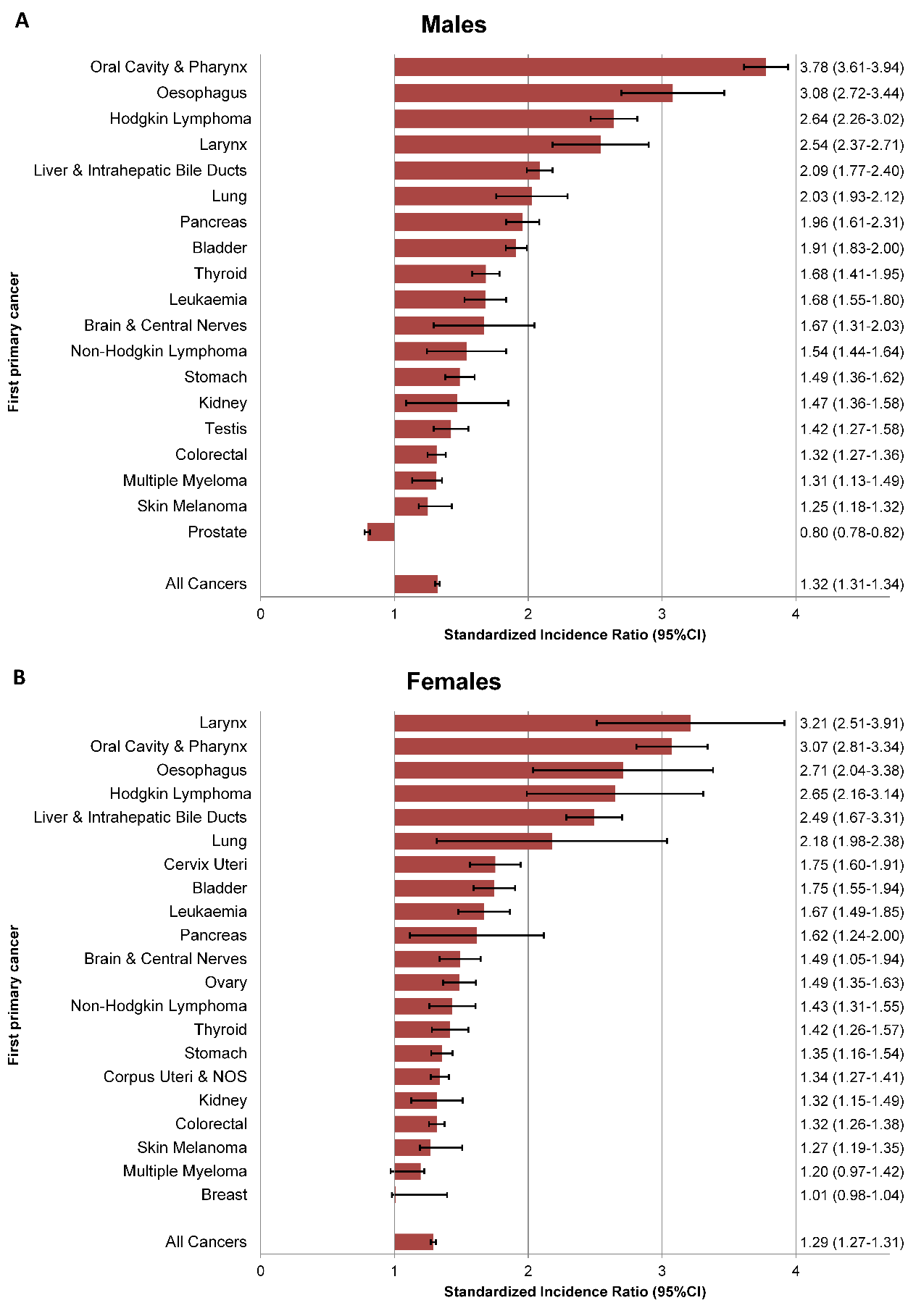
**

**Figure S1: Relative risk of second primary cancer by site of first primary cancer and sex including synchronous tumours including the first six month after initial diagnosis for risk calculations**
